# The prevalence and correlates of common mental disorders among adolescents and young adults in rural South Africa: analysis of household survey data informed by lived experience experts

**DOI:** 10.1186/s12889-025-23890-y

**Published:** 2025-08-22

**Authors:** Audrey Moyo, Lovemore Nyasha Sigwadhi, Stanley Carries, Nokwanda Sithole, Reuben Christopher Moyo, Arvin Bhana, Peter Suwirakwenda Nyasulu, Eugene Lee Davids, Theresa Munyombwe, Innocent Maposa, Darshini Govindasamy

**Affiliations:** 1 Division of Epidemiology and Biostatistics, Department of Global Health, Faculty of Medicine and Health Sciences, Stellenbosch University, Cape Town, South Africa; 2 Health Systems Research Unit, South African Medical Research Council, Cape Town, South Africa; 3 Department of Psychology, Faculty of Humanities, University of Pretoria, Pretoria, South Africa; 4 Leeds Institute of Cardiovascular and Metabolic Medicine, University of Leeds, Leeds, UK; 5 Leeds Institute for Data Analytics, University of Leeds, Leeds, UK; 6 Centre for Research in Health Systems, University of KwaZulu-Natal, Durban, South Africa

**Keywords:** Common mental disorders, Depressive symptoms, Anxiety symptoms, Adolescents, Young adults, Lived experience

## Abstract

**Background:**

Depression and anxiety are among the most common mental disorders (CMDs) affecting adolescents and young adults (AYA). CMDs often emerge during adolescence and young adulthood, yet are frequently diagnosed in adulthood. Understanding the burden and correlates of CMDs is essential for informing public mental health interventions. However, limited research incorporates the perspectives of AYA with lived experience of mental health conditions. This study aimed to determine the prevalence and factors associated with CMDs among AYA aged 15–24 years in South Africa, incorporating insights from those with lived experience.

**Methods:**

First wave South African Population Research Infrastructure Network (SAPRIN) mental health data, collected between March 2021 and April 2022 in three rural areas, were used. AYA with complete mental health information were included. CMDs were the primary outcome, defined as the presence of either depressive symptoms (Patient Health Questionnaire-2 [PHQ-2] score ≥ 3), anxiety symptoms (Generalised Anxiety Disorder-2 [GAD-2] score ≥ 3), or both. Participatory workshops with 17 AYA aged 18–30 years with lived experience of mental health conditions informed the secondary data analysis and interpretation. Exposure variables included age, sex, living with both parents, relationship to the household head, school attendance, and occupation. Multivariable logistic regression was used to examined correlates of CMDs, accounting for clustering by surveillance site.

**Results:**

Among *n* = 11,346 AYA, CMD prevalence was 9.0% (*n* = 1026). Increased odds of CMDs were associated with having children (adjusted odds ratio [aOR] = 1.27; 95% confidence interval [CI]: 1.04–1.54, *p* = 0.016), being the head of household, a spouse, or a sibling of the household head (aOR = 1.56; 95% CI: 1.29–1.89; *p* < 0.001), and being an extended family member of the household head (aOR = 1.26; 95% CI: 1.08–1.46; *p* = 0.002).

**Conclusion:**

CMD prevalence among rural AYA in South Africa was 9.0%, with early parenthood and not being the child of the household head identified as key correlates of increased CMD risk. These findings suggest that early parenthood and family structure may contribute to CMDs. Future research should explore the long-term impacts of these correlates using longitudinal datasets, while considering broader contextual factors.

**Supplementary Information:**

The online version contains supplementary material available at 10.1186/s12889-025-23890-y.

## Key message

A significant proportion of rural AYA report experiencing CMDs, with early parenthood and not being the child of the household head identified as key risk factors. These findings underscore the need for community-and-school-based social support interventions, alongside economic empowerment initiatives, to address the mental health needs of this population.

**Supplementary Information:**

The online version contains supplementary material available at 10.1186/s12889-025-23890-y.

## Introduction

 Mental disorders contribute to 15.0% of the total disease burden among adolescents globally [1]. A study using data from the 2019 Global Burden of Disease study reported prevalence rates of 13.9% among those aged 15–19 years and 13.6% among those aged 20–24 years [2]. Among these, depression and anxiety are the most common mental disorders (CMDs). An estimated 3.5% of adolescents aged 15–19 years experience depression, while 5.5% experience anxiety [1]. CMDs often emerge during mid-to-late adolescence and young adulthood (15–24 years) but are frequently first diagnosed in adulthood [3]. The lifetime risk of CMD ranges between 10.4% and 20.6% [4], with long-term consequences, including recurrence, substance use, suicide, and impaired social and physical functioning [5, 6].

In South Africa, recent studies estimate the prevalence of depressive symptoms among adolescents and young adults (AYA) to be 30.3% [7], while anxiety symptoms affect 74.2% [8]. However, these estimates were based on studies conducted in specific subpopulations rather than community-based samples. For instance, one study assessed depressive symptoms among female AYA aged 15–24 years from six South African provinces (*n* = 515) [7], a group that may report higher CMD prevalence compared to the general population due to gender-related factors [9]. Another study reported a high prevalence of anxiety symptoms among university students with a mean age of 22 years from eight South African provinces (*n* = 1411) [8], a population that may encounter unique academic and social stressors not representative of the broader AYA population.

Previous studies from high-income and low-and-middle income countries have found demographic and socioeconomic factors, including age, sex, family structure, having children, education attendance, and occupation to be associated with CMDs, including depression and anxiety among AYA [10–14]. For instance, AYA from single-parent households are more likely to experience depressive and anxiety symptoms [15]. Furthermore, the challenges of early parenthood and the pressures of balancing education or employment have been shown to contribute to CMDs [16, 17]. However, most studies are based on peri-urban samples, and the factors impeding mental health in rural settings may be quite unique, given the limited access to health and social services [18] and the high burden of HIV and non-communicable disease (NCDs) in these areas [19]. Understanding these correlates is essential for informing community-based screening and linkage to mental health care services, particularly equipping lay counsellors with tools to identify at-risk AYA and connect them to appropriate support. The prevalence of CMDs in these settings is also critical for directing mental health programme budgets.

Despite growing recognition of the correlates of CMDs, the perspectives of AYA with lived experience of mental health remain underrepresented in research. Lived experience refers to the firsthand knowledge gained from past or present mental health challenges [20]. AYA with lived experience, known as lived experience experts (LEEs), self-identify as such and are not required to have a professional diagnosis or prior engagement with mental healthcare services [20]. LEEs are not study participants, but contribute as investigators, collaborators, advisors, or workshop participants, offering insights that inform research design, planning, and implementation [20]. Although LEEs’ involvement in mental research is increasingly recognised, their engagement in study design and data interpretation remains limited [21]. Involving LEEs allows for advocacy, addressing unmet needs, and highlighting mental health inequalities [21]. To address this gap, this study engaged LEEs in workshop discussions to inform the selection of correlates of CMDs and interpretation of findings in secondary data analysis.

This study aims to determine the prevalence of CMDs and their correlates, incorporating lived experience insights to enhance the interpretation of findings for community-based mental health interventions targeting rural AYA. This approach bridges the gap between research and real-world application, fostering effective interventions.

## Method

### Study design, setting, and participants

Secondary data from the first wave of the South African Population Research Infrastructure Network (SAPRIN) Mental Health Data Prize (MHDP) 2022 longitudinal study were used [22]. The SAPRIN MHDP2022 comprises data collected across three rural Health and Demographic Surveillance System (HDSS) sites (referred to as nodes by SAPRIN) in South Africa: the MRC/Wits University Agincourt HDSS located in Bushbuckridge District, Mpumalanga; the University of Limpopo DIMAMO HDSS in the Capricorn District, Limpopo; and the Africa Health Research Institute (AHRI) in uMkhanyakude District, KwaZulu-Natal province. These HDSS sites conduct regular longitudinal surveillance of over 75,000 households (320,000 residents). Surveillance has been ongoing since 1993. SAPRIN collects demographic, health, and socioeconomic information from the whole population residing in these surveillance sites [23].

Data collection in these HDSS sites were collected through interviews with household proxy respondents, typically the head of household or a senior household member. Data collection methods evolved from paper-based interviews to computer-assisted personal and telephonic interviews. Since 2019, data collection was conducted in three rounds annually, combining in-person and telephonic methods to ensure regular intervals of data collection. Each HDSS site collected data using different questionnaires, and SAPRIN harmonised the resulting datasets before them publicly available.

During the COVID-19 surveillance, mental health questionnaires were administered to household respondents, with a total of 90,000 interviews conducted by the end of 2021 across households in the three HDSS sites. The component of the mental health questionnaires administered are outlined in section 7.2 and 7.3 of the Individual Health Module in the SAPRIN study protocol [24]. A detailed description of the variables collected at SAPRIN HDSS sites is available in another study [23].

This study used SAPRIN MHDP 2022 individual-level data collected from 1 March 2021 to 30 April 2022 [25–27]. The dataset included 34,715 observations of AYA aged 15–24 years, among which 11 participants had two recorded visits. For those individuals, the most recent observation was selected, except in one case where the most recent visit contained missing data, In that instance, the earlier complete observation was used. After applying this criterion, a total of 11,346 AYA aged 15–24 years who provided complete responses to the mental health questionnaires were included in the study. The median age of the participants was 19 years (Interquartile range [IQR] = 17–22 years), and the majority were female (*n* = 5720, 50.4%). Most participants were from a surveillance site in KwaZulu-Natal province (*n* = 4839, 42.6%), followed by those from a surveillance site in Limpopo province (*n* = 4035, 35.6%).

### Lived experience workshop

To inform the secondary data analysis, insights were obtained from a lived experience workshop conducted on 13 October 2022. The workshop included 17 LEEs (9 females and 8 males), aged 18–30 years, from KwaZulu-Natal province. Participants were either receiving professional healthcare for mental health symptoms or had overcome symptoms in the past year. Discussions were facilitated in English and the local language (isiZulu) by trained fieldworkers, with LEEs recruited from clinics, youth mental health non-governmental organisations (NGOs), and ongoing research studies. Discussions explored perceptions of a good life, factors contributing to mental health challenges, barriers to treatment access, and coping mechanisms. Insights from these discussions informed the selection of exposure variables for this study. Some key quotes from the discussions are provided in Table S2 in the Supplementary Material 1.

### Measures

#### Outcome(s)

CMDs were the primary outcome in this study, defined as the presence of at least one of the following: depressive or anxiety symptoms. Depressive symptoms were assessed using the two-item Patient Health Questionnaire (PHQ-2), with a score ≥ 3 indicating probable depression. Anxiety symptoms were assessed using two item Generalized Anxiety Disorder (GAD-2), with a score ≥ 3 indicating probable anxiety [28]. The PHQ-2 and GAD-2 scales have been validated in South African primary healthcare settings as screening measures for probable depression and anxiety [28]. The PHQ-2 includes two key questions: “Over the past two weeks, how often have you been bothered by (i) having little interest or pleasure in doing things? and (ii) feeling down, depressed, or hopeless?” [29]. The GAD-2 includes two key questions: “Over the past two weeks, how often have you been bothered by (i) feeling nervous, anxious or on edge? and (ii) not being able to stop or control worrying?” [30]. Responses for scales are 0 = “not at all”, 1 =” several days”, 2 = “more than half the days”, and 3= “nearly every day”. Total scores range from 0 to 6, obtained by summing responses for the two questions in each scale. A score of ≥ 3 on either the PHQ-2 or GAD-2 indicated the presence of depressive or anxiety symptoms [29, 30]. In this study, the PHQ-2 and GAD-2 scales exhibited high internal reliability, with Cronbach’s alpha values of 0.96 and 0.97 respectively.

#### Exposure(s)

The selection of exposure variables for this study was informed by consultations with AYA with lived experience of mental health challenges. Key stressors on mental health identified during these discussions were grouped into three themes: financial instability, family instability, and young parenthood (Table S2 in Supplementary Material 1). Accordingly, variables from the SAPRIN dataset were selected to reflect these lived experiences, to the extent permitted by available data.

To capture financial instability, we included *current school attendance* (not attending or ≤ Grade 7, and Grade 8–12), and *occupation* (unemployed, and studying or employed). These variables reflect LEEs’ concerns around financial insecurity, joblessness, and disrupted educational pathways.

To reflect family instability and young parenthood, we included *living with parents* (neither parent, mother only, father only, and both parents), *relationship to the head of the household* (child; head, spouse, or sibling; extended family; and non-relatives), and *ever having children* (no, and yes).

Demographic variables included *age* (15–19 years and 20–24 years) and *self-identified sex* (male and female). For a more detailed description of the variables, refer to Table S1 in Supplementary Material 1.

### Statistical analysis

Datasets were cleaned and processed for analysis, including the selection of key variables, handling missing data, and identification of participants with complete responses to the PHQ-2 and GAD-2 for inclusion. Descriptive statistics, including frequencies and proportions, were used to summarise the characteristics and prevalence of CMDs among AYA. Multivariable logistic regression models were applied to estimate the associations between exposure variables and CMDs, including exposures with a significance of *p* < 0.15. Models were adjusted for age and sex and accounted for clustering by surveillance site using the ‘*miceadds’* package in R. Adjusted odds ratios (aORs) with 95% confidence intervals (CIs) were reported for multivariable models. Statistical significance was set at p < 0.05. Sensitivity analyses were performed. First, the demographic characteristics of participants included and excluded from the study were compared to assess potential selection bias. Second, the impact of including the variable *living with both parents* in the parsimonious model was examined to assess the robustness of observed associations. All analyses were conducted using RStudio version 4.4.0 [31].

### Missing data

Missing data on exposures were addressed by sourcing information from previous waves of data and using multiple imputation by chained equations (MICE). Most exposures had missingness of less than 5%, except for current school attendance (19.2%) and occupation (27.1%). Missing values for self-identified sex, living with parents, ever had children, and relationship to head of household were imputed using historical data from waves between 1999 and 2020. MICE was applied to the variables current school attendance and occupation. For exposures with less than 30% missing data, MICE was used, as it has demonstrated robustness in handling missingness of up to 50% [32]. Five imputations were performed using the *‘mice’* R package, with a seed set to ensure replicability. Pooled estimates were utilised for logistic regression. These methods were employed for the main analysis.

### Ethics considerations

Secondary ethics approval for this study was obtained from the South African Medical Research Council (SAMRC) Human Research Ethics Committee (HREC) (protocol: EC001-01/2023) and Stellenbosch University HREC (project: 31003). The study uses secondary data collected by SAPRIN, which received ethics approval from the SAMRC HREC (protocol EC010-3/2021) before conducting surveillances. In addition, separate ethical approvals were obtained for each HDSS surveillance site. The Agincourt surveillance site was approved by the University of Witwatersrand HREC (protocol: M190305), AHRI surveillance site was approved by the University of Kwazulu-Natal Biomedical Research Ethics Committee (protocol: BE290/16), and DIMAMO surveillance site was approved by the University of Limpopo Turfloop Research Ethics Committee (protocol: TREC/116/2018).

Written informed consent was obtained from all participants during the primary data collection for SAPRIN and its HDSS sites. Participants consented to their anonymised data being shared for scientific analysis through the SAPRIN data repository. For minors aged 15–17 years, informed consent was obtained from parents or legal guardians, while adolescents provided assent [24]. However, adolescents aged 15–17 years who were married, parents, or living in child-headed households were considered emancipated minors and provided independent consent, in accordance with ethical guidelines. Additionally, informed consent and signed confidentiality agreements were obtained from participants for engagement with LEEs to ensure the protection of personal information.

This research was conducted in accordance with the Declaration of Helsinki.

## Results

### Participant characteristics

Of the 34,704 AYA aged 15–24 years, 11,346 participants with complete CMD information (that is, complete responses to both PHQ-2 and GAD-2 items) were included, while 23,358 (67.3%) were excluded due to incomplete outcome data. Table 1 shows that most participants were aged 15–19 years (*n* = 5874, 51.8%), female (*n* = 5720, 50.4%), had never had children (*n* = 9202, 81.1%), were attending or had completed grades 8–12 (*n* = 8779, 77.4%), and were unemployed (*n* = 5079, 44.8%). Nearly half lived with only their mothers (*n* = 5101, 45.0%), while 22.3% (*n* = 2529) lived with both parents, and 5.3% (*n* = 603) lived with their fathers only. The majority were children of the head of the household (*n* = 5888, 51.9%), whilst 29.2% (*n* = 3310) were extended family members and 11.8% (*n* = 1339) were head, spouse, or siblings of the household head. Missing data were observed for self-identified sex, living with both parents, ever had children, relationship to head of household (each *n* = 481, 4.2%), and current school attendance (*n* = 2180, 19.2%) and occupation (*n* = 3079, 27.1%).

**Table 1:** Participant characteristics and CMD prevalence by participants characteristics, SAPRIN MHDP 2022

Characteristic	Overall (*n* = 11,346)	With CMD (*n* = 1026)	With no CMD (*n* = 10,320)	*p*-value
	*n* (%)	*n* (%)	*n* (%)	
Age (years)				0.420
15–19	5874 (51.8%)	544 (9.3%)	5330 (90.7%)	
20–24	5472 (48.2%)	482 (8.8%)	4990 (91.2%)	
Median (IQR)	19 (17–22)			
Self-identified sex				0.143
Male	5145 (45.3%)	451 (8.8%)	4694 (91.2%)	
Female	5720 (50.4%)	549 (9.6%)	5171 (90.4%)	
* Missing*	*481 (4.2%)*	*26 (5.4%)*	*455 (94.6%)*	
Living with both parents				0.011
Neither parent	2632 (23.2%)	262 (10.0%)	2370 (90.0%)	
Mother only	5101 (45.0%)	490 (9.6%)	4611 (90.4%)	
Father only	603 (5.3%)	57 (9.5%)	546 (90.5%)	
Both parents	2529 (22.3%)	191 (7.6%)	2338 (92.4%)	
* Missing*	*481 (4.2%)*	*26 (5.4%)*	*455 (94.6%)*	
Ever had children				0.019
No	9202 (81.1%)	821 (8.9%)	8381 (91.1%)	
Yes	1663 (14.7%)	179 (10.8%)	1484 (89.2%)	
* Missing*	*481 (4.2%)*	*26 (5.4%)*	*455 (94.6%)*	
Relationship with household head				< 0.001
Child	5888 (51.9%)	467 (7.9%)	5421 (92.1%)	
Head, spouse, or sibling	1339 (11.8%)	158 (11.8%)	1181 (88.2%)	
Extended family	3310 (29.2%)	328 (9.9%)	2982 (90.1%)	
Non-relatives	328 (2.9%)	47 (14.3%)	281 (85.7%)	
* Missing*	*481 (4.2%)*	*26 (5.4%)*	*455 (94.6%)*	
Current school attendance				0.266
Not attending or ≤ Grade 7	387 (3.4%)	47 (12.1%)	340 (87.9%)	
Grade 8–12	8779 (77.4%)	900 (10.3%)	7879 (89.7%)	
* Missing*	*2180 (19.2%)*	*79 (3.6%)*	*2101 (96.4%)*	
Occupation				0.829
Unemployed	5079 (44.8%)	440 (8.7%)	4639 (91.3%)	
Studying/employed	3188 (28.1%)	271 (8.5%)	2917 (91.5%)	
* Missing*	*3079 (27.1%)*	*315 (10.2%)*	*2764 (89.8%)*	

### CMD prevalence and participant characteristics

Out of the of 11,346 AYA, 9.0% (*n* = 1026) had CMD, reporting either depressive symptoms, anxiety symptoms, or both (Table S2 in Supplementary Material 1). Table 1 shows that CMDs were slightly more prevalent among participants aged 15–19 years (*n* = 544, 9.3%), females (*n* = 549, 9.6%), those who had children (*n* = 179, 10.8%), those with ≤ Grade 7 education or not attending school (*n* = 47, 12.1%), and the unemployed (*n* = 440, 8.7%). Participants living with neither parent (*n* = 262, 10.0%) had the highest prevalence of CMDs, followed by those living with their mothers only (*n* = 490, 9.6%), whilst those living with both parents (*n* = 191, 7.6%) had the lowest. Similarly, CMDs were most prevalent among AYA who were not related to the household head (*n* = 47, 14.3%). The separate prevalence of depressive and anxiety symptoms by participant characteristics is presented in Table S4 in Supplementary Material 1.

### Factors associated with CMD

Table 2 presents the associations between exposure variables and CMD. Participants who had children had 27.1% (aOR = 1.271; 95% CI: 1.046–1.544, *p* = 0.016) higher odds of reporting at least one CMD compared to those who never had children. AYA who were heads, spouses or siblings of the household head (aOR = 1.560; 95% CI: 1.290–1.886; *p* < 0.001), extended family members (aOR = 1.256; 95% CI: 1.084–1.455; *p* = 0.002), and non-relatives (aOR = 1.914; 95% CI: 1.386–2.642; *p* < 0.001) had higher odds of reporting CMD compared to those who were children of the household head. Age, self-identified sex and current school attendance were not significantly associated with CMD. However, the ORs indicate that AYA aged 20–24 years (aOR = 0.924; 95% CI: 0.806–1.059; *p* = 0.257) and those attending grade 8–12 (aOR = 0.780; 95% CI: 0.576–1.058; *p* = 0.110) had lower odds of reporting CMD compared to their respective reference categories, while females (aOR = 1.067; 95% CI: 0.926–1.231; *p* = 0.370) had slightly higher odds. Living with parents and occupation were excluded from the parsimonious model, as they were not significant in the model selection process. Similar results were observed for the separate logistic regression models for depressive and anxiety symptoms (Table S6 and S7 in Supplementary Material 1).

**Table 2:** Univariate and multivariable logistic regression model of CMD association with demographic and socioeconomic variables among AYA

Characteristic	Univariate model		Multivariable model	
	OR (95% CI)	*p*-values	aOR (95% CI)	*p*-values
Age (years)				
15–19 (ref)				
20–24	0.946 (0.832, 1.076)	0.401	0.924 (0.806, 1.059)	0.257
Self-identified sex				
Male (ref)				
Female	1.119 (0.984, 1.273)	0.087	1.067 (0.926, 1.231)	0.370
Ever had children				
No (ref)				
Yes	1.247 (1.052, 1.478)	0.011*	1.271 (1.046, 1.544)	0.016*
Relationship with household head				
Child (ref)				
Head, spouse, or sibling	1.557 (1.288, 1.883)	< 0.001*	1.560 (1.290, 1.886)	< 0.001*
Extended family	1.259 (1.088, 1.456)	0.002*	1.256 (1.084, 1.455)	0.002*
Non-relatives	1.927 (1.396, 2.659)	< 0.001*	1.914 (1.386, 2.642)	< 0.001*
Current school attendance				
Not attending or ≤ grade 7 (ref)				
Grade 8–12	0.770 (0.571, 1.038)	0.086	0.780 (0.576, 1.058)	0.110

*Ref* reference group, *OR* odds ratio, *aOR* adjusted odds ratio, *CI* confidence interval

*p < 0.05

### Sensitivity analysis

Participants with incomplete information on the CMD outcome were slightly older (median age: 20 years; IQR: 18–22) and more likely to be male (*n* = 10,937, 46.8%) compared to those included in the final analysis (Table S5 in Supplementary Material 1). In univariate analysis, AYA living with both parents (OR = 0.765; 95% CI: 0.629–0.929) were less likely to experience CMDs compared to those living with neither parent. However, this association reduced and became non-significant in the multivariate model. Living with mother only (aOR = 1.195; 95% CI: 1.006–1.420) emerged as a significant correlate of CMD risk after adjustment (Table S8 in Supplementary Material 1).

## Discussion

This study used a large rural population-based dataset to examine the prevalence and factors associated with CMDs among AYA aged 15–24 years in South Africa. It also incorporated insights from LEEs’ participatory workshop discussions to inform the selection of variables for analysis and interpretation of findings. The findings indicate that 9.0% of participants reported at least one CMD (either depressive or anxiety symptoms). The prevalence of CMDs was 9.3% among 15–19-year-olds and 8.8% among 20–24-year-olds. Factors significantly associated with CMDs included having children, and being the head, spouse or sibling, extended family member or non-relative of the household head.

The overall CMD prevalence of 9.0% in this study is lower than estimates from previous South African studies conducted among young women and university students, and peri-urban adolescents [7, 8, 33]. However, the use of a large, rural population-based sample may provide a more comprehensive representation of AYA in these settings. The lower prevalence could reflect underreporting due to fear of stigmatisation surrounding mental health in rural areas [34]. In an already resource-constrained setting [35], stigma may lead AYA to withhold information due to fear of judgement. Furthermore, the slightly lower prevalence among young adults may indicate differences in exposure to risk factors, coping mechanisms, or healthcare-seeking behaviours, warranting further research.

AYA who had children had significantly higher odds of reporting CMDs, a trend observed in other South African studies [36, 37]. Early parenthood is associated with increased financial strain, disrupted education, and challenges in securing stable employment, all of which are established risk factors for poor mental health [16]. Moreover, young parents may face social stigma, reduced autonomy, and greater caregiving burdens, contributing to CMDs [16]. Similarly, AYA who were immediate family members, extended family members, or non-relatives of the household head had higher odds of CMDs. This may reflect increased household responsibilities, economic pressure, or relational stress within the family unit [38]. Research suggests that living with both biological parents is associated with better mental health outcomes, whereas alternative living arrangements may result in reduced financial and emotional support, as well as strained family dynamics, increasing CMD risk [38].

These findings of this study strongly align with insights from LEEs, who highlighted financial instability, lack of parental support, young parenthood, and strained family relationships as major stressors contributing to CMDs (Table S3 in Supplementary Material 1). These experiences directly correspond with the observed higher odds of CMDs among AYA with children and those living outside nuclear family structures. This reinforces the need for mental health interventions that strengthen social support networks, particularly for AYA experiencing family instability or financial hardship. Evidence suggests that supportive relationships from family, peers, and schools serve as protective factors against poor mental health outcomes [39]. LEEs similarly emphasised the value of community-based support, with some describing the need for accessible, government-supported centres that provide safe spaces for youth engagement, skill-building, and psychosocial support (Table S2 Supplementary Material 1). Therefore, interventions should prioritise peer support programmes, school-based mental health services, and community-led initiatives to provide psychosocial support for vulnerable AYA, including young parents and those lacking family support systems. Schools can play a critical role in fostering resilience by offering mental health education, counselling services, and peer mentorship initiatives to promote mental well-being [40].

### Limitations

This study used a large, population-based dataset from the first wave of a longitudinal study in South Africa. This allowed comprehensive analysis of CMDs in a large rural sample. Compared to previous studies using smaller, selective samples [7, 8], we observed lower prevalence of CMDs, highlighting the value of large-scale data in capturing broader population trends. The integration of insights from LEEs also strengthened the interpretation of findings, ensuring that results are grounded in real-world experiences of AYA facing mental health challenges.

However, several limitations must be considered. First, this study focused on rural populations across three provinces, limiting the generalisability of findings to urban population and the country as a whole. While the results may be relevant to other rural populations in low- and middle-income countries, they do not capture the unique mental health dynamics of urban settings. Engagement with LEEs was limited to per-urban AYA from existing studies, and the LEEs’ age range (18–30 years) was slightly older than most survey participants (15–19 years). This age difference may mean that some factors endorsed by LEEs are applicable to older AYA in urban settings, potentially limiting the applicability of these insights to broader populations. Second, the study used cross-sectional data from the first wave of a longitudinal dataset, precluding causal inferences. Future research using longitudinal datasets could provide a clearer understanding of the temporal relationships between CMD correlates and outcomes. Third, the analysis was restricted to variables with less than 30% missingness, leading to exclusion of some household-level variables that may have been relevant to understanding AYA mental health. In addition, for certain variables with missing data, historical information from 1999 to 2020 was used to supplement missing values. This may have introduced classification bias, as participant characteristics could have changed in 2022. Moreover, the data were collected during the COVID-19 pandemic, a period marked by widespread disruption to education, employment, and social systems that disproportionately affected young people [41]. While these circumstances likely influenced AYA mental health, COVID-19-specific impacts were not explicitly examined in this analysis, potentially limiting interpretation of some findings within the pandemic context. Fourth, although we evaluated potential confounders, residual confounding remains a possibility, as some unmeasured factors may still influence CMD risk [42]. Fifth, a large number of AYA were excluded from this study due to missing information on the CMD outcome. Those included were slightly younger and more likely to be female, which suggests that older AYA and males may be underrepresented in the findings. This selection bias may limit the generalizability of the results to these subgroups. Finally, while substantial evidence links poor mental health in AYA to contextual factors such as food insecurity, exposure to violence, and social and cultural norms [7, 43], these variables were not captured in the SAPRIN dataset therefore could not be examined in this analysis. Notably, literature also acknowledges factors such as the high burden of HIV and NCDs, as well as limited access to health services as key barriers to mental health in rural settings [18, 19]. Future research should aim to incorporate these critical contextual factors and broader social determinants of mental health to provide a more comprehensive understanding of CMDs.

## Conclusion

The prevalence of CMDs (either depressive or anxiety symptoms) was 9.0% among AYA aged 15–24 years, with early parenthood and not being a biological or foster child of the household head identified as key correlates. These findings suggest that childcare burdens and weaker household support structures contribute to CMD risk. The strong alignment between these results and insights from LEEs underscores the need for targeted mental health interventions for AYA experiencing financial hardship, family instability, and childcare responsibilities. School- and community-based programmes are recommended to strengthen social support networks, expand access to mental health services, and promote peer support initiatives. Furthermore, economic empowerment interventions targeting young parents and financially vulnerable AYA may help mitigate CMD risk. Future research should employ longitudinal datasets to assess the long-term health impacts of early parenthood, family structure, and socioeconomic stressors, as well as explore unmeasured contextual factors.

## Supplementary Information

Supplementary Material 1.

## Data Availability

The dataset used in this study was obtained from the SAPRIN data repository and can be requested from the data managers via the repository.
