# Supplementary material for: The prevalence and correlates of common mental disorders among adolescents and young adults in rural South Africa: analysis of household survey data informed by lived experience experts
Source: BMC Public Health. 2025 Aug 22;25:2900. doi: 10.1186/s12889-025-23890-y (PMC12372366; doi:10.1186/s12889-025-23890-y)
Supplement: Supplementary file 1 — Supplementary Material 1. [file 12889_2025_23890_MOESM1_ESM.docx]

**Table S1: Description of variables**

| **Variables** | **Survey questions/description** | **Survey response options** | **Grouping and recoding** |
| --- | --- | --- | --- |
| **Age** | Respondent’s age at the time of survey, computed from the date of birth. | **Format:** dd-mm-yyyy  **Range:** 29-04-1996 to 01-01-2006 | **Age groups:**  0 = 15-19 years  1 = 20-24 years |
| **Self-identified sex** | Respondent’s self-identified sex. | 1 = Male  2 = Female | No recoding needed |
| **Surveillance site** | SAPRIN Health and Demographic Surveillance System (HDSS) sites | 1 = Agincourt (Mpumalanga)  2 = DIMAMO (Limpopo)  3= AHRI (KwaZulu-Natal) | No recoding needed |
| **Living with both parents** | Whether the respondent lived with their parents at the time of the survey.  Defined from:   - Mother’s status (unknown, coresident, alive, dead) - Father’s status (unknown, coresident, alive, dead) | 0 = Unknown  1 = Coresident  2 = Alive  3 = Dead | 0 = Neither parent  1 = Mother only  2 = Father only  3 = Both parents |
| **Ever had children** | Number of children ever born to respondent. | Continuous [Min 0, Max 4] | 0 = No (0 children)  1 = Yes (>=1 children) |
| **Relationship with household head** | Respondent’s relationship to the head of the household at the time of survey. | 1 = Head  2 = Spouse  3 = Child  4 = Son/daughter- in-law  5 = Grandchild  6 = Parent  7 = Parent-inlaw  8 = Grandparent  9 = Sibling  10 = Other relative  11 = Domestic worker/tenant  12 = Unrelated/other | **0 = Child** (respondent is the child or foster child of the household head)  **1 = Head, spouse, or sibling** (respondent is the household head, their spouse, or sibling)  **2 = Extended family** (respondent is a grandchild, in-law, or other relative of the household head)  **3 = Non-relatives** (domestic worker, tenant, unrelated individual, or unrelated) |
| **Current school attendance** | Whether the respondent was attending school at the time of survey. | -1 = Unknown  0-12 = Grade 0-12  96 = Refused  97 = Not applicable  98 = No education 99 = Not attending, 999 = Missing | 0 = Not attending or ≤ Grade 7  1 = Grade 8-12, |
| **Occupation** | Whether the respondent was employed or studying at the time of survey. | 1 = Studying  2 = Looking for work  3 = Retired, 4 = Sick/injured  5 = Pregnant/childcare  6 = Other childcare  7 = Care for sick/injured  8 = Retrenched  9 = Nothing  10 = Other  11 = Don’t know  12 = Refused  14 = Disabled  15 = Employed  16 = Part-time employment  17 = Pensioner  18 = Not looking  19 = Household duties/care  21 = Unemployment  999 = Missing | 0 = Unemployed  1 = Studying or employed (full-time, part-time) |

**Table S2: Key quotes from LEEs on challenges affecting mental health**

| **Theme** | **Key quotes** |
| --- | --- |
| **Financial instability and unemployment** | *“Being jobless”* (P16)  *“Lack of finances”* (P2)  *“Insecurity and lack of employment”* (P5) |
| **Lack of parental support, family instability, and young parenthood** | *“Family issues”* (P6)  *“Having an alcoholic parent also leads to poverty because they use all the money towards alcohol and not accordingly”* (P8)  *“Not all of us were able to complete schooling; not all of us after being pregnant were able to find employment and not all of us have single parents who are employed and are able take care; for example; I am born of a single parent who is unemployed and because of that I am hindered from completing my schooling and my mother has had to make a plan raising us and getting us to school which in itself leads to poverty”* (P7)  “Being a young parent” (P7)  *“Having people; maybe four or five that depend on you as a breadwinner, and you end up not able to provide for yourself which may lead to depression”* (P4) |
| **The role of community support** | *“If there were centres that the government could provide for communities; like this centre; we are currently in where the community would receive help for free; that would be great”* (P20  *“If we have facilities like this one it would encourage us to make use of our gifts and talents and this will help me not to be exposed to the streets and as a girl it would prevent me on the chances of getting pregnant because of my engagements in the activities”* (P4) |

**Table S3: CMD prevalence among AYA aged15-24 years, SAPRIN MHDP 2022**

|  | **n (%)** |
| --- | --- |
| **CMD** |  |
| With no CMD | 10,320 (91.0%) |
| With CMD | 1026 (9.0%) |
| **Individuals with CMD (n=1026)** |  |
| With anxiety symptoms only | 67 (6.5%) |
| With depressive symptoms only | 170 (16.6%) |
| With both anxiety and depressive symptoms | 789 (76.9%) |
|  |  |
| **Depressive symptoms** |  |
| With no depressive symptoms | 10,387 (91.5%) |
| With depressive symptoms | 959 (8.5%) |
|  |  |
| **Anxiety symptoms** |  |
| With no depressive symptoms | 10,490 (92.5%) |
| With depressive symptoms | 856 (7.5%) |

**Table S4: Prevalence of depressive and anxiety symptoms by participants’ characteristics, SAPRIN MHDP 2022**

| **Characteristic** | **With depressive symptoms (n = 1026)** | **With no depressive symptoms (n = 10,320)** | **p-value** | **With anxiety symptoms (n = 1026)** | **With no anxiety symptoms (n = 10,320)** | **p-value** |
| --- | --- | --- | --- | --- | --- | --- |
|  | **n (%)** | **n (%)** |  | **n (%)** | **n (%)** |  |
| **Age (years)** |  |  | 0.380 |  |  | 0.602 |
| 15-19 | 510 (8.7%) | 5364 (91.3%) |  | 451 (7.7%) | 5423 (92.3%) |  |
| 20-24 | 449 (8.2%) | 5023 (91.8%) |  | 405 (7.4%) | 5067 (92.6%) |  |
| **Self-identified sex** |  |  | 0.198 |  |  | 0.589 |
| Male | 423 (8.2%) | 4722 (91.8%) |  | 386 (7.5%) | 4759 (92.5%) |  |
| Female | 511 (8.9%) | 5209 (91.1%) |  | 446 (7.8%) | 5274 (92.2%) |  |
| *Missing* | *25 (5.2%)* | *456 (94.8%)* |  | *24 (5.0%)* | *457 (95.0%)* |  |
| **Living with both parents** |  |  | 0.037 |  |  | 0.024 |
| Neither parent | 240 (9.1%) | 2392 (90.9%) |  | 213 (8.1%) | 2419 (91.9%) |  |
| Mother only | 455 (8.9%) | 4646 (91.1%) |  | 415 (8.1%) | 4686 (91.9%) |  |
| Father only | 57 (9.5%) | 546 (90.5%) |  | 46 (7.6%) | 557 (92.4%) |  |
| Both parents | 182 (7.2%) | 2347 (92.8%) |  | 158 (6.2%) | 2371 (93.8%) |  |
| *Missing* | *25 (5.2%)* | *456 (94.8%)* |  | *24 (5.0%)* | *457 (95.0%)* |  |
| **Ever had children** |  |  | 0.032 |  |  | 0.069 |
| No | 768 (8.3%) | 8434 (91.7%) |  | 686 (7.5%) | 8516 (92.5%) |  |
| Yes | 166 (10%) | 1497 (90%) |  | 146 (8.8%) | 1517 (91.2%) |  |
| *Missing* | *25 (5.2%)* | *456 (94.8%)* |  | *24 (5.0%)* | *457 (95.0%)* |  |
| **Relationship with household head** |  |  | <0.001 |  |  | <0.001 |
| Child | 437 (7.4%) | 5451 (92.6%) |  | 392 (6.7%) | 5496 (93.3%) |  |
| Immediate family | 148 (11.1%) | 1191 (88.9%) |  | 127 (9.5%) | 1212 (90.5%) |  |
| Extended family | 305 (9.2%) | 3005 (90.8%) |  | 277 (8.4%) | 3033 (91.6%) |  |
| Non-relatives | 44 (13.4%) | 284 (86.6%) |  | 36 (11%) | 292 (89%) |  |
| *Missing* | *25 (5.2%)* | *456 (94.8%)* |  | *24 (5.0%)* | *457 (95.0%)* |  |
| **Current school attendance** |  |  | 0.392 |  |  | 0.697 |
| Not attending or ≤ grade 7 | 43 (11.1%) | 344 (88.9%) |  | 36 (9.3%) | 351 (90.7%) |  |
| Grade 8-12 | 848 (9.7%) | 7931 (90.3%) |  | 755 (8.6%) | 8024 (91.4%) |  |
| *Missing* | *68 (3.1%)* | *2112 (96.9%)* |  | *65 (3.0%)* | *2115 (97.0%)* |  |
| **Occupation** |  |  | 0.428 |  |  | 0.740 |
| Unemployed | 413 (8.1%) | 4666 (91.9%) |  | 368 (7.2%) | 4711 (92.8%) |  |
| Studying/employed | 243 (7.6%) | 2945 (92.4%) |  | 224 (7%) | 2964 (93%) |  |
| *Missing* | *303 (9.8%)* | *2776 (90.2%)* |  | *264 (8.6%)* | *2815 (91.4%)* |  |

**Table S5: Participant characteristics for AYA with incomplete CMD information, SAPRIN MHDP 2022**

| **Characteristic** | **With missing CMD (n = 23,358)** |
| --- | --- |
|  | **n (%)** |
| **Age (years)** |  |
| 15-19 | 10520 (45.0%) |
| 20-24 | 12838 (55.0%) |
| Median (IQR) | 20 (18-22) |
| **Self-identified sex** |  |
| Male | 10937 (46.8%) |
| Female | 10116 (43.3%) |
| *Missing* | *2305 (9.9%)* |
| **Living with both parents** |  |
| Neither parent | 5844 (25.0%) |
| Mother only | 8804 (37.7%) |
| Father only | 1138 (4.9%) |
| Both parents | 5266 (22.5%) |
| *Missing* | *2306 (9.9%)* |
| **Ever had children** |  |
| No | 18683 (80.0%) |
| Yes | 2371 (10.2%) |
| *Missing* | *2304 (9.9%)* |
| **Relationship with household head** |  |
| Child | 11715 (50.2%) |
| Immediate family | 1488 (6.4%) |
| Extended family | 7385 (31.6%) |
| Non-relatives | 466 (2.0%) |
| *Missing* | *2304 (9.9%)* |
| **Current school attendance** |  |
| Not attending or ≤ grade 7 | 626 (2.7%) |
| Grade 8-12 | 13395 (57.3%) |
| *Missing* | *9337 (40.0%)* |
| **Occupation** |  |
| Unemployed | 8870 (38.0%) |
| Studying/employed | 8921 (38.2%) |
| *Missing* | *5567 (23.8%)* |

**Table S6: Univariate and multivariable logistic regression model of depressive symptoms association with demographic and socioeconomic variables among AYA,**

| **Characteristic** | **Univariate Model** | | **Multivariate Model** | |
| --- | --- | --- | --- | --- |
|  | **OR (95% CI)** | **p-values** | **aOR (95% CI)** | **p-values** |
| **Age (years)** |  |  |  |  |
| 15-19 (ref) |  |  |  |  |
| 20-24 | 0.940 (0.823, 1.073) | 0.362 | 0.916 (0.796, 1.055) | 0.223 |
| **Self-identified sex** |  |  |  |  |
| Male (ref) |  |  |  |  |
| Female | 1.107 (0.970, 1.265) | 0.133 | 1.055 (0.911, 1.222) | 0.475 |
| **Ever had children** |  |  |  |  |
| No (ref) |  |  |  |  |
| Yes | 1.233 (1.034, 1.470) | 0.019* | 1.264 (1.033, 1.546) | 0.023* |
| **Relationship with household head** |  |  |  |  |
| Child (ref) |  |  |  |  |
| Immediate family | 1.540 (1.266, 1.873) | <0.001* | 1.543 (1.269, 1.877) | <0.001* |
| Extended family | 1.248 (1.074, 1.450) | 0.004* | 1.244 (1.069, 1.448) | 0.005* |
| Non-relatives | 1.913 (1.374, 2.664) | <0.001* | 1.901 (1.364, 2.647) | <0.001* |
| **Current school attendance** |  |  |  |  |
| Not attending or ≤ grade 7 (ref) |  |  |  |  |
| Grade 8-12 | 0.822 (0.588, 1.148) | 0.251 | 0.838 (0.597, 1.175) | 0.306 |
| Ref: reference group; OR: odds ratio; aOR: adjusted odds ratio; CI: confidence interval; *p < 0.05 | | | | |

**Table S7: Univariate and multivariable logistic regression model of anxiety symptoms association with demographic and socioeconomic variables among AYA,**

| **Characteristic** | **Univariate Model** | | **Multivariate Model** | |
| --- | --- | --- | --- | --- |
|  | **OR (95% CI)** | **p-values** | **aOR (95% CI)** | **p-values** |
| **Age (years)** |  |  |  |  |
| 15-19 (ref) |  |  |  |  |
| 20-24 | 0.961 (0.836, 1.105) | 0.577 | 0.940 (0.811, 1.089) | 0.410 |
| **Self-identified sex** |  |  |  |  |
| Male (ref) |  |  |  |  |
| Female | 1.059 (0.921, 1.218) | 0.423 | 1.005 (0.861, 1.174) | 0.945 |
| **Ever had children** |  |  |  |  |
| No (ref) |  |  |  |  |
| Yes | 1.208 (1.003, 1.454) | 0.046* | 1.257 (1.017, 1.554) | 0.034* |
| **Relationship with household head** |  |  |  |  |
| Child (ref) |  |  |  |  |
| Immediate family | 1.470 (1.194, 1.810) | <0.001* | 1.471 (1.194, 1.812) | <0.001* |
| Extended family | 1.259 (1.076, 1.474) | 0.004* | 1.260 (1.075, 1.476) | 0.004* |
| Non-relatives | 1.709 (1.192, 2.450) | <0.001* | 1.704 (1.188, 2.444) | 0.004* |
| **Current school attendance** |  |  |  |  |
| Not attending or ≤ grade 7 (ref) |  |  |  |  |
| Grade 8-12 | 0.878 (0.607, 1.270) | 0.491 | 0.898 (0.618, 1.304) | 0.571 |
| Ref: reference group; OR: odds ratio; aOR: adjusted odds ratio; CI: confidence interval; *p < 0.05 | | | | |

**Table S8: Sensitivity analysis assessing the impact of living with both parents on univariate and multivariable logistic regression models examining associations between CMDs and its correlates among AYA,**

| **Characteristic** | **Univariate Model** | | **Multivariate Model** | |
| --- | --- | --- | --- | --- |
|  | **OR (95% CI)** | **p-values** | **aOR (95% CI)** | **p-values** |
| **Age (years)** |  |  |  |  |
| 15-19 (ref) |  |  |  |  |
| 20-24 | 0.946 (0.832, 1.076) | 0.401 | 0.935 (0.814, 1.074) | 0.340 |
| **Self-identified sex** |  |  |  |  |
| Male (ref) |  |  |  |  |
| Female | 1.119 (0.984, 1.273) | 0.087 | 1.064 (0.923, 1.228) | 0.391 |
| **Living with both parents** |  |  |  |  |
| Neither parent (ref) |  |  |  |  |
| Mother only | 0.995 (0.851, 1.163) | 0.947 | 1.195 (1.006, 1.420) | 0.042* |
| Father only | 0.987 (0.732, 1.331) | 0.934 | 1.222 (0.897, 1.666) | 0.203 |
| Both parents | 0.765 (0.629, 0.929) | 0.007* | 1.051 (0.828, 1.335) | 0.683 |
| **Ever had children** |  |  |  |  |
| No (ref) |  |  |  |  |
| Yes | 1.247 (1.052, 1.478) | 0.011* | 1.272 (1.047, 1.546) | 0.015* |
| **Relationship with household head** |  |  |  |  |
| Child (ref) |  |  |  |  |
| Immediate family | 1.557 (1.288, 1.883) | <0.001* | 1.649 (1.328, 2.048) | <0.001* |
| Extended family | 1.259 (1.088, 1.456) | 0.002* | 1.286 (1.085, 1.524) | 0.004* |
| Non-relatives | 1.927 (1.396, 2.659) | <0.001* | 1.973 (1.409, 2.764) | <0.001* |
| **Current school attendance** |  |  |  |  |
| Not attending or ≤ grade 7 (ref) |  |  |  |  |
| Grade 8-12 | 0.770 (0.571, 1.038) | 0.086 | 0.808 (0.581, 1.125) | 0.208 |
| Ref: reference group; OR: odds ratio; aOR: adjusted odds ratio; CI: confidence interval; *p < 0.05 | | | | |
